# Costing of three feeding regimens for home-based management of children with uncomplicated severe acute malnutrition from a randomised trial in India

**DOI:** 10.1136/bmjgh-2017-000702

**Published:** 2018-03-06

**Authors:** Charu C Garg, Sarmila Mazumder, Sunita Taneja, Medha Shekhar, Sanjana Brahmawar Mohan, Anuradha Bose, Sharad D Iyengar, Rajiv Bahl, Jose Martines, Nita Bhandari

**Affiliations:** 1 International Consultant and Visiting Professor, Institute for Human Development, New Delhi, India; 2 Centre for Health Research and Development, Society for Applied Studies, New Delhi, India; 3 Department of Community Health, Christian Medical College, Vellore, Tamil Nadu, India; 4 Research and Evaluation Department, Action Research and Training for Health, Udaipur, Rajasthan, India; 5 Department of Maternal, Newborn, Child and Adolescent Health, World Health Organization, Geneva, Switzerland; 6 Centre for Intervention Science in Maternal and Child Health, Centre for International Health, University of Bergen, Bergen, Norway

**Keywords:** costs, severe acute malnutrition (SAM), community management, India, child

## Abstract

**Trial design:**

Three feeding regimens—centrally produced ready-to-use therapeutic food, locally produced ready-to-use therapeutic food, and augmented, energy-dense, home-prepared food—were provided in a community setting for children with severe acute malnutrition (SAM) in the age group of 6–59 months in an individually randomised multicentre trial that enrolled 906 children. Foods, counselling, feeding support and treatment for mild illnesses were provided until recovery or 16 weeks.

**Methods:**

Costs were estimated for 371 children enrolled in Delhi in a semiurban location after active survey and identification, enrolment, diagnosis and treatment for mild illnesses, and finally treatment with one of the three regimens, both under the research and government setting. Direct costs were estimated for human resources using a price times quantity approach, based on their salaries and average time taken for each activity. The cost per week per child for food, medicines and other consumables was estimated based on the total expenditure over the period and children covered. Indirect costs for programme management including training, transport, non-consumables, infrastructure and equipment were estimated per week per child based on total expenditures for research study and making suitable adjustments for estimations under government setting.

**Results:**

No significant difference in costs was found across the three regimens per covered or per treated child. The average cost per treated child in the government setting was estimated at US$56 (<3500 rupees).

**Conclusion:**

Home-based management of SAM with a locally produced ready-to-use therapeutic food is feasible, acceptable, affordable and very cost-effective in terms of the disability-adjusted life years saved and gross national income per capita of the country. The treatment of SAM at home needs serious attention and integration into the existing health system, along with actions to prevent SAM.

**Trial registration number:**

NCT01705769; Pre-results.

**Key questions**

**What is already known about this topic?**

- Evidence suggests community-based therapeutic care was more cost-effective as compared with costs for inpatient treatment.
- However, controversy prevails over the cost-effectiveness of domiciliary treatment, particularly the ready-to-use therapeutic food component of the management strategy, which is deemed to be expensive and logistically difficult to procure, distribute and sustain.

**What are the new findings?**

- The study provides an estimate of the costs per child treated (US$56) and overall costs required to cover a population with known incidence, for the first time in India for home-based management of uncomplicated SAM.
- A randomised multicenter trial shows no conclusive evidence to prefer one regimen over the other in terms of costs per child treated.
- Home based management of SAM requires approximately 20% in administrative costs,10% for the cost of screening and identification, 5% for the cost of peer support, and 60-65% for the cost of treatment.
- Different level of health system development, types of terrain and and socio-cultural settings impacts the costs of home-based management of uncomplicated SAM.

**Recommendations for policy**

- Low costs of treatment of uncomplicated SAM in community based settings suggests shifting the treatment from institutional care to home-based care.
- Screening and identification can be introduced as a preventive care strategy at primary care settings.

## Introduction

India accounts for over half the global burden of severe acute malnutrition (SAM) in the world.^1^ Before 2007, WHO recommended that all children with SAM be treated as inpatients in hospitals and fed milk-based diets (F75 followed by F100). This facility-based management comprised the initial stabilisation phase lasting 2–7 days, followed by a management phase lasting for several weeks.^2^ However, most children with SAM do not receive facility-based management due to several constraints, such as insufficient number of beds, lack of trained inpatient staff, costs of management, iatrogenic infections in already immunocompromised children, and direct and indirect costs to families due to prolonged hospital stay, inability to take care of health and home, and loss of wages. Since 2007, WHO has recommended ready-to-use therapeutic food (RUTF) for home-based management of uncomplicated SAM.^3^ Acceptance of this recommendation is low due to lack of evidence from controlled trials of RUTF efficacy compared with other treatment options, and the dilemma and controversy around using commercial preparations of RUTF over locally produced indigenous RUTF in India, which may be less expensive and more sustainable if proven efficacious.^4^

It is imperative therefore that adequate evidence is generated on the efficacy of home-based management of uncomplicated SAM and identify the most appropriate diet regimen and management strategy. Domiciliary treatment may be preferred by families over facility care due to reduced opportunity costs.^1 5^ Additionally home treatment is likely to reduce the burden on the health system by restricting admission to only children with complicated SAM. Inpatient treatment is resource-intensive requiring skilled staff, with the number of cases often going beyond the capacity of inpatient facilities.^1^

Within this context, we conducted a randomised trial to compare the efficacy of a centrally produced RUTF (RUTF-C) and locally prepared RUTF (RUTF-L) for home-based management of children with uncomplicated SAM on recovery rates compared with micronutrient-enriched (augmented), energy-dense, home-prepared food (A-HPF), which was the comparison group.^6^ This study was conducted in Delhi, Udaipur and Vellore and showed that RUTF-L is more efficacious than A-HPF at home. The recovery rates with RUTF-L, RUTF-C and A-HPF were 56.9%, 47.5% and 42.8%, respectively. This is the first randomised trial confirming that RUTF-L is more efficacious than A-HPF at home.

However, the question arises on the cost of treatment and the scale-up and policy implications for the government. Controversy prevails over the cost-effectiveness of domiciliary treatment, particularly the RUTF component of the management strategy, which is deemed to be expensive and logistically difficult to procure, distribute and sustain.^7–10^ There are few studies that have analysed the cost-effectiveness of community management of SAM (CMAM). An Ethiopian study shows that institutional cost per child treated was US$262, while community-based therapeutic care was half the cost, US$128.^11^ Community-based strategy was found to be cost-effective in a Bangladesh study, US$26 per disability-adjusted life year (DALY) compared with US$1344 per DALY for inpatient treatment.^12^ This evidence suggests that CMAM is cost-effective.

We conducted costing analysis as one of the secondary objectives of the main trial^6^ to estimate the cost of home-based management of children suffering from uncomplicated SAM, where treatment was delivered at home. Costs were calculated per week per child for activities under research setting and estimated for the activities that are likely to be done under the government setting. In this paper we describe the interventions that were costed, analyse the costs, and compare the implementation costs and costs of the three feeding regimens per child in the main trial.

## Methods

The main trial was an individually randomised multicentre trial conducted between October 2012 and April 2015, and enrolled 906 children aged 6–59 months with uncomplicated SAM. Foods, counselling and feeding support were provided until recovery or 16 weeks. Outcomes were measured weekly during the treatment phase. In the sustenance phase access to the government nutrition services was facilitated. The primary outcome of the main trial was recovery during treatment phase, weight for height (WFH) ≥−2 SD and absence of oedema in the feet. The trial was conducted in the urban slums and resettlement colonies in the national capital region of Delhi; rural and predominantly tribal areas in Udaipur, Rajasthan; and rural and semiurban areas in Vellore, Tamil Nadu, where the prevalence of SAM is above the national average of 6.4%. Children with WFH <−3 SD of the WHO standard, and/or oedema of both feet, or both were included. Children with complicated SAM were referred to a hospital. Complications included presence of signs of severe illness, lethargic or unconscious, unable to drink or breast feed, vomits everything, convulsions, bulging fontanelle or stiff neck, pneumonia defined as fast breathing, chest indrawing, stridor, crepitations or bronchial breathing on auscultation, diarrhoea with dehydration, severe anaemia defined as haemoglobin <6, and other IMNCI danger signs. The cost of inpatient treatment was excluded from this study. The costs of three feeding regimens—RUTF-C, RUTF-L and A-HPF—were determined for each activity undertaken for enrolled children only under the treatment phase for the Delhi site. For Udaipur and Vellore sites, detailed costs were not calculated and only the key differences were identified. Detailed methods, primary and secondary outcomes, sample size estimation, randomisation, allocation concealment, blinding, and statistical analyses for primary and secondary outcomes with additional analyses are described in the main paper.^6^

### Interventions/major activity and subactivities

Survey and identification were undertaken in the community setting by Accredited Social Health Activist (ASHA)-like workers (ALW) in a research setting. The activities included moving from house to house, confirming date of birth, filling in the register, measuring mid-upper arm circumference (MUAC) following written informed consent, filling in and recording the data, providing Integrated Management of Neonatal and Childhood Illness (IMNCI) guidelines to children with MUAC >13 cm or for families who refuse treatment with MUAC <13 cm, and escorting children with MUAC <13 cm to clinics.

Screening and enrolment were done at the community clinic, which would be equivalent to a primary healthcare centre in the government setting. Children were screened for SAM using the WFH criteria by the outcome measurement (OM) worker in a research setting, who is equivalent to the auxiliary nurse midwife (ANM) in the government setting. The physician/nutritionist (ANM in the government setting) administered the HemoCue and appetite tests and treated children for minor ailments. Additional research activities included obtaining written informed consents, obtaining mothers’ weight and height, escorting the child to the hospital and randomising the child to one of the three study regimens. In Delhi, 371 children were enrolled in the study and were randomised into one of the three feeding regimens, 124 each under RUTF-C and RUTF- L and 123 under A-HPF.

Physicians or nurse managed minor illnesses. Cointerventions were administered; these were amoxicillin for 5 days for all children enrolled, mebendazole for children over 2 years for 3 days, and two bottles of iron to be consumed over 60 days for children over 2 years in the A-HPF group, with anaemia. Nutritionists counselled about the regimens, what to feed, how to feed, good hygiene and breastfeeding practices. In the research setting, home visits were also made for counselling, which may not happen in the government setting.

During the first week, children randomised to one of the three regimens were provided specific food according to their weight, and the mothers were counselled on feeding by the nutritionists. Additional research activities included home visits to observe feeding, taking the dietary recall, filling in the baseline form, taking consent for assisted feeding and introducing peer counsellors to the families by nutritionists or ALW follow-up workers (ALW-FU). Under A-HPF it also included demonstrating recipes, and providing milk and egg vouchers.

Weekly management under the three regimens for weeks 2–16 included recording weight, height and MUAC; checking oedema in the feet and skinfold thickness by the OM staff (ANM in the government setting); replenishing supplies by storekeeper, which would be equivalent to a pharmacist in the government setting; and counselling by nutritionists, who are equivalent to ANMs in the government setting. Additional research activities included travel to the house of the child for anthropometric measurements and visit by the ALW-FU worker to take dietary recall, observe feeding, collect used jars and replenish supplies.

During weeks 2–16, children with illnesses were referred to the study clinic and treated by the study physician. For the research component of costing, the costs of visiting the households and costs of consultation over the phone were included. It also included emergency treatment and referral if required, assessment on completion of the treatment, and introducing to the Anganwadi worker.

Costs for supervised feeding included costs paid to peer supporters and telephone reimbursement. The cost was incurred over 14 months for 182 enrolled children as this activity was initiated later into the study.

### Data

Data on the number of covered and treated children for each activity were collected. Of the 48 634 covered children aged 6–59 months identified during survey, 371 children with SAM were treated. Table 1 provides information on major activities, number of covered and treated children for each activity, human resources and time taken for each activity, and consumables used per week in the research setting and the estimated resources that would be used in the government setting. Information was collected on salaries of the providers and administrative staff under the research setting and the equivalent in the government setting; expenditures incurred for the non-consumables, such as equipment used for preparation of RUTF-L, communication and data collection equipment such as phones, tablets and others, furniture, and computers and printers; training costs that took into consideration the duration, people trained and cost of trainers; the costs of communication and transportation services especially for outreach activities; and the number of weeks of treatment under each regimen.

**Table 1:** Average time taken for each activity and population covered for government programme and research

Major activities	Children (n)	Average time taken in minutes for each provider		Consumables under each activity
		Under research (R)	For government (G)	
1. Survey and identification	House visits: 185 500 Surveyed/covered: 48 634 –Identified with potential risk (<13 cm MUAC): 2861	2 ALWs: 29 each	ASHA: 25	Survey register, MUAC tape, stationary (included under the overall administrative costs)
2. Screening and enrolment	Screened: 2902 HemoCue and appetite tests: 546 Minor treatment: 1328 Enrolled/treated: 371	Physician: 46 Nutritionist: 91 OM team (2): 9 each ALW-FU: 30 Escort team (2): 100	Physician: 33 Nurse: 56 ANM: 7	eeZee Paste, medicines for minor ailments
3. Management through cointervention and counselling	Counselled and given amoxicillin: 371 Mebendazole: 162 Iron: 102	Physician: 17 Nurse/nutritionist: 18	Physician: 17 Nurse/nutritionist: 18	Amoxicillin, mebendazole, iron tablets
4.1 Management of RUTF-C regimens during week 1	Children counselled and provided RUTF-C: 124	Storekeeper: 13 Nutritionist: 48 ALW-FU: 45	Pharmacist: 13 Nutritionist: 14	eeZee Paste, containers
4.2. Management of RUTF-L regimens during week 1	Children counselled and provided RUTF-L: 124	Storekeeper: 17 Nutritionist: 48 ALW-FU: 40	Pharmacist: 17 Nutritionist/nurse: 12	Sugar, skimmed milk powder, peanut paste, vitamin, soya bean and sunflower oil, mineral mix, and jars
4.3. Management of A-HPF regimens during week 1	Children counselled and provided A-HPF: 123	Storekeeper: 24 Nutritionist: 63 ALW-FU: 88	Pharmacist: 24 Nutritionist/nurse: 10	Pulses, rice, oil and sugar; eggs and milk; jars and utensils; and Ricona LP bottle
5.1. Weekly management and follow-up of child on RUTF-C	Children treated: 124	OM staff: 55 ALW-FU: 12 Storekeeper: 13	Nurse: 27 Pharmacist: 6	eeZee Paste containers
5.2. Weekly management and follow-up of child on RUTF-L	Children treated: 124	OM staff: 55 ALW-FU: 12 Storekeeper: 17	Nurse: 27 Pharmacist: 6	Sugar, skimmed milk powder, peanut paste, vitamin, soya bean and sunflower oil, mineral mix, and jars
5.3. Weekly management and follow-up of child on A-HPF	Children treated: 123	OM staff: 55 ALW-FU: 13 Storekeeper: 29	Nurse: 27 Pharmacist: 8	Pulses, rice, oil and sugar; eggs and milk; jars and utensils; and Ricona LP bottle
6. Diagnosis and treatment during treatment phase: weeks 2–16	Consultation in clinic: 371 On phone: 157 Home: 14 Emergency: 102	Physician in clinic: 30 Consultation over phone: 10 Home visits: 55 ALW: 120 min to escort the child to clinic	Physician in clinic only: 30	Medicines for minor ailments
7. Peer support with supervision for feeding*	Children treated through peer support: 182	Peer supporter and supervisors: costs 132 rupees per treated child per week	ASHA: cost per treated child: 150 rupees^13^	

*The costs under research were per week and government costs were per child followed and obtaining MUAC below a certain level. If treatment is over 10 weeks on average, the government costs will be 150/10 or 15 rupees per week much lower than research.

A-HPF, augmented, energy-dense, home-prepared food; ALW, ASHA-like worker; ALW-FU, ALW follow-up worker; ANM, auxiliary nurse midwife; ASHA, Accredited Social Health Activist; MUAC, mid-upper arm circumference; OM, outcome measurement; RUTF-C, centrally produced ready-to-use therapeutic food; RUTF-L, locally produced ready-to-use therapeutic food.

The number of children screened is higher than those identified under risk during survey as there were walk-ins as well.

### Cost estimation

Direct costs were estimated per week per child for human resources and consumables in rupees for the treatment under three regimens. The average exchange rate (US$1=62 rupees) of 2013 and 2014 was used to convert all the costs into dollars (http://www.xe.com/currencyconverter/).

The human resources costs were calculated per week per child based on the salaries and time taken for each subactivity defined in table 1. A time motion type of study was used to collect the average time spent by a provider for each activity. If a subactivity required two or three providers, then the weighted average of their per cent allocated time was taken, where weight was the percentage of children treated under each provider for a given subactivity.

The costs of food, medicines and other consumables given in table 1 were estimated for the entire period of the research study and based on the number of children covered for each activity, for the treatment under three regimens. The costs of consumables under screening and enrolment were calculated for (1) costs of eeZee Paste (RUTF-C produced by Compact Foods Ltd. India) for appetite test per child, which were estimated at US$0.05 (3.5 rupees); and (2) costs of medicines for treating minor ailments per child, which were estimated at US$0.03 (1.9 rupees) (obtained from the total costs of medicines provided over 25 months for 1328 children).

For management with cointerventions and counselling, the weighted cost of amoxicillin tablets given thrice a day to 371 treated children for 5 days, and mebendazole syrup given twice a day to 162 children more than 2 years of age for 3 days, was calculated at US$0.45 (27.1 rupees) per child.

The average cost per week per child for the RUTF-C regimen (including eeZee Paste and containers given to 124 children over 25 months) was estimated at US$0.44 ((357110×12)/(25×52×124)=26.6 rupees) under research activity and US$0.40 (24.25 rupees) under the government programme (assuming no costs for containers).

The costs of the RUTF-L regimen were calculated by adding the (1) costs of the consumables, (2) annualised costs (depreciated over 5 years) of equipment to prepare the paste and (3) costs of two staff for 27 months who prepared the paste and packaged them. Costs per week per child were estimated at US$1.4 (84 rupees) under research activity and US$0.7 (43 rupees) under the government programme (assuming lower costs of containers and staff).

The costs per week per child under A-HPF, calculated by adding the costs of raw materials, Riconia LP bottle and jars provided to 123 children over 25 months, were estimated at US$0.9 (770 860×12/(25×52×123)= 57.9 rupees) under research and US$0.9 (56.8 rupees) under the government programme (assuming that the jars and utensils were not included).

During the treatment phase, the costs of medicines were calculated for 1328 children and estimated at US$0.03 (1.85 rupees) per child per week for diagnosis and treatment. Further, under A-HPF, besides the regimen, iron supplementation was also given after the first 2 weeks of treatment to 102 children, and the cost per child per week was estimated at US$0.2 (14.32 rupees).

Under the research programme US$11 (700 rupees) was planned per week for each peer supporter who was identified from the community to support the mother in feeding the child. The costs per week per child incurred for 182 children over 14 months were US$2.2 (132 rupees) per treated child per week and US$0.01 (0.5 rupees) per covered child per week. Under the government programme, ASHA is given an incentive of US$2.5 (150 rupees) for each child with SAM treated or reaching an MUAC of 125 mm.^13^ Hence, under the government programme, the average cost for peer support per covered child was calculated at US$0.08 (150×371/48 635=1.1 rupees) for any regimen.

Indirect costs such as administrative and programme costs cannot be allocated for specific child but are required for overall implementation of the programme. These costs were incurred over 33 months on the following:

1. Personnel for administration, management and data support activities, which were computed from full-time equivalent (FTE) (based on the number of staff and the percentage of time each staff devoted) for the staff such as managers, supervisors, quality control officers, office staff and attendants for administration and management. The total administrative costs were estimated at US$11063 (686 000 rupees) for research and US$1246 (77300 rupees) for government programme. Government costs have lower salaries for the staff and lower estimated FTE.
2. Training in MUAC measurement, field support, counselling and standardisation of anthropometry equipment. The total training costs were estimated at US$3717 (230 500 rupees) for research and US$1083 (67 200 rupees) for government programme (training is assumed only at the start of the programme and is calculated using government salaries for one staff of each type).
3. Transport, which included costs for one vehicle for the referred patients and for the survey team under research. For the government programme these costs were calculated based on 102 children who needed referral transport. It was assumed that each of these children was paid US$5 (300 rupees) (same as the amount paid for transportation for institutional delivery in government programme). The total transport costs were estimated at US$4107 (254 700 rupees) for research and US$493 (30 600 rupees) for government programme.
4. Stationary and computers included office supplies, field supplies, printing and computer supplies. Over 80% of these costs were considered for purely research purposes (not for programme activities) and not included. The total costs were estimated at US$3193 (198 000 rupees) for research and US$422 (26 100 rupees) for government programme (excluding computer supplies).
5. Non-consumable goods, which included mainly anthropometry equipment, were calculated using annual 20% depreciation. The costs under both research and government programme were estimated at US$1330 (82 500 rupees).
6. Infrastructure and equipment. Only 5% of the rental for the field office including security, water, electricity and office maintenance/repair costs were included under the research programme and were estimated at US$2344 (145 400 rupees). For the government programme these costs were considered to be a part of the health system.
7. Communication, which included the costs of phones and net books for data collection, were calculated after depreciating the total expenditures for equipment at 33%. Only 40% of the total costs were included under the research programme and were estimated at US$1145 (71016 rupees). For government no expenditure was added under this head.

The programme management, administrative, training and operations costs were estimated at US$26935 (1670 000 rupees) for the research and US$4564 (283 000 rupees) for government programme over a period of 33 months. The costs were allocated equally over all the children covered and treated under the programme and were estimated at US$0.6 (34.3 rupees) for research and US$0.1 (5.8 rupees) for government per covered child per week, and at US$72 (4496 rupees) and US$12 (765 rupees) for research and government programme, respectively, per treated child per week.

The average recovery rate for each of the three regimens was calculated by taking the weighted average of the number of weeks taken for child to recover where weights were the number of children recovered under different weeks of follow-up - from week one to week 16. The average weeks of treatment under the three regimens were 10.3 weeks for RUTF-C, 9.6 weeks for RUTF-L and 11 weeks for A-HPF.

## Results

The baseline characteristics of enrolled children, the results of the primary and secondary outcomes, and additional analyses are described in the main paper.^6^ For the entire period of 33 months over which the research activity was conducted, 48 634 children were covered and 371 children were treated, with 124 under each of the RUTF-C and RUTF-L regimens, and 123 under the A-HPF regimen. For each activity, the costs of consumables are added to the human resources costs to determine the total costs per week per child for staff and consumables. The results are presented in table 2 for research and government separately for treated and covered children.

**Table 2:** Average human resource and consumable costs per treated and covered child for each major activity in US dollars

.	Activities	Average costs per week per treated child		Children treated (n)	Average costs per week per covered child		Children covered (n)
		Research	Government programme		Research	Government programme	
1	Survey and identification	51.6	3.8	371	0.387	0.029	48 634
2	Screening and enrolment	10.4	2.9	371	0.079	0.023	48 634
3	Management through cointervention and counselling at time of enrolment	1.9	1.4	371	0.015	0.010	48 634
	Management of regimens during first week of enrolment						
4	RUTF-C	2.1	0.9	124	0.005	0.002	48 634
5	RUTF-L	2.9	1.2	124	0.007	0.003	48 634
6	A-HPF	3.3	1.5	123	0.008	0.004	48 634
	Per week costs for weeks 2–16 (treatment phase)						
7	Diagnosis and treatment for mild illnesses for children with SAM during treatment phase	4.1	2.6	371	0.031	0.020	48 634
8	Weekly follow-up of child on RUTF-C	3.0	0.6	124	0.016	0.002	48 634
9	Weekly follow-up of child on RUTF-L	3.9	0.9	124	0.018	0.003	48 634
10	Weekly follow-up of child on A-HPF	3.5	1.1	123	0.017	0.004	48 634

A-HPF, augmented, energy-dense, home-prepared food; RUTF-C, centrally produced ready-to-use therapeutic food; RUTF-L, locally produced ready-to-use therapeutic food; SAM, severe acute malnutrition.

During week 1, the costs under each regimen included the cost of survey and identification, screening and enrolment, management through cointervention and counselling at time of enrolment, and management of each of the regimens. During the treatment phase, costs per week included diagnosis and treatment and weekly follow-up, including measurement of length/height and weight to ascertain recovery of children under each of the three regimens. Table 3 presents the human resources and consumable costs under these three regimens for week 1 and weeks 2–16 and also the costs of the regimen per week per covered and treated child.

**Table 3:** Human resources and consumables costs per covered and treated child per week for each of the 3 regimens in US dollars

Regimen	Week 1		Weeks 2–16		Costs of regimen per week per child	
	Research	Government programme	Research	Government programme	Research	Government programme
Costs of covered children						
RUTF-C	0.493	0.064	0.047	0.022	0.001	0.001
RUTF-L	0.495	0.065	0.050	0.023	0.003	0.002
A-HPF	0.496	0.065	0.049	0.024	0.002	0.002
Costs of treated children						
RUTF-C	66.1	8.9	7.1	3.2	0.4	0.4
RUTF-L	66.9	9.3	8.0	3.5	1.4	0.7
A-HPF	67.2	9.5	7.5	3.7	0.9	0.9

A-HPF, augmented, energy-dense, home-prepared food; RUTF-C, centrally produced ready-to-use therapeutic food; RUTF-L, locally produced ready-to-use therapeutic food.

We find the cost per week per covered child was lowest for RUTF-C and highest for A-HPF under the government programme. Under the research activity, during the first week A-HPF costs were higher than RUTF-L and RUTF-C, as more time was spent on demonstration of recipes, providing the feed and setting up the system of milk and egg vouchers with local persons. During weeks 2–16, the RUTF-L cost per week was higher under the research activity, as the cost of RUTF-L preparation was higher. Around US$66 (4100 rupees) was spent during week 1 per treated child under research activity, and between US$9 (550 rupees) and US$10 (600 rupees) under the government programme.

The total costs for covered and treated children were derived based on the average number of weeks that a given regimen was required for the treatment. These were estimated at 10.3, 9.6 and 11 weeks for RUTF-C, RUTF-L and A-HPF, respectively, as shown in table 4. The costs of peer support were based on the number of weeks of treatment and were added to the total human resources and consumable costs. Further administrative costs per child were added to the total costs calculated for human resources, drugs, consumables and peer supporters. An equal number of covered children (48 634/3) was assumed to be covered across the three regimens for administrative costs.

**Table 4:** Total costs for covered and treated children under three regimens including the cost of peer support and administration in US dollars

Regimen	Average number of weeks treatment given	Average costs of peer support per covered child in the population		Total HR and consumable costs per child for complete treatment, including peer support/follow-up		Administrative costs per child in the population		Total costs per child for complete treatment, including HR and consumables, peer support, and administrative costs		Total costs for all children for complete treatment, including peer support/follow-up and administrative costs	
		Research	Government programme	Research	Government programme	Research	Government programme	Research	Government programme	Research	Government programme
Costs of covered child											
RUTF-C	10.3	0.1	0.02	1.0	0.3	0.6	0.1	1.6	0.4	25 430	6185
RUTF-L	9.6	0.1	0.02	1.0	0.3	0.6	0.1	1.6	0.4	25 181	6074
A-HPF	11	0.1	0.02	1.1	0.3	0.6	0.1	1.6	0.4	26 309	6734
Costs of treated children											
RUTF-C	10.3	22	2	154	41	73	12	227	53	28 127	6613
RUTF-L	9.6	21	2	157	42	73	12	229	54	28 439	6746
A-HPF	11	23	2	166	49	73	12	238	61	29 311	7533

A-HPF, augmented, energy-dense, home-prepared food; HR, human resource; RUTF-C, centrally produced ready-to-use therapeutic food; RUTF-L, locally produced ready-to-use therapeutic food.

Table 4 shows that the total costs per covered child were estimated at US$1.6 for each of the three regimens under research setting, and US$0.4 under the government programme. The total costs for 48 634 covered children were estimated at US$76 920 (4769 000 rupees) for research and US$18 993 (1178 000 rupees) under the government programme. The total costs under the government programme varied from US$6074 (377 000 rupees) to US$6734 (418 000 rupees) across the three regimens, as shown in column 7 of table 4. The lowest cost was for the RUTF-L, followed by RUTF-C and then A-HPF. Even though the costs per week per child were slightly higher for RUTF-L as compared with RUTF-C in table 3, the total cost for a treatment per child per episode is lowest for RUTF-L as the number of weeks of treatment was lowest for this regimen. The high costs of A-HPF may also be attributed to excess ration that was provided to the distressed family over and above the requirement of the child.

The average costs per treated child per episode under research were estimated between US$227 and US$238 (14 063–14 775 rupees) for each of the three regimens. Out of the total costs per treated child, about 30% of the costs were for administration under research activity. For the government programme, the costs per treated child were between US$53 and US$61 (3307–3797 rupees) for each of the three regimens, with administrative costs at about 20% of the total costs. The research costs were higher due to higher salaries, more staff being used for the same activity and also more activities being included under those costs such as escorts to the hospital or taking informed consent. The total costs for treating 371 children were estimated at US$85 876 (5324 000 rupees) for research programme and US$20 892 (1295 000 rupees) for the government programme.

## Discussion

### Comparison with other studies and interpretation

Our results show that the government will require just about US$0.4 (25 rupees) per covered child in the defined population or under US$61 (3782 rupees) per treated child per episode for children with SAM. Under the research activity with higher costs of human resources, training and administration, the costs of treatment per child with SAM in community setting were under US$ 238. The results of our analysis compare well with that found in other studies. In African studies the cost of ambulatory community-based treatment of SAM ranged between US$46 and US$ 453 per child.^14^ The key component of the cost of community-based management of SAM is the cost of RUTF. However, if produced locally with local ingredients, the costs are substantially reduced. Additionally in facilities, staff time and health service costs were attributed to the higher cost of facility-based management. Household or parental costs are lower than the health service costs.^14^ A Malawi study showed that implementing CMAM is within the highly cost-effective gross national income (GNI) per capita threshold of US$250.^15^ In Zambia, the cost of community-based management of SAM was US$203 per case treated, US$1760 per life saved and US$53 for DALY averted.^16^ In Zambia, of the total cost of US$203, the cost of RUTF was 36%, health centre visit cost 13%, hospital admission cost 17% and technical support while establishing the programme was 34%.^16^ In Ethiopia, the mean cost per child treated was US$284 in the facility and US$134 in the community.^11^ The institutional cost per child treated was US$262 in the facility and US$128 in the community. Out of the institutional costs in the facility, 46.6% were personnel, while in the community the major (43.2%) cost was that of RUTF. The opportunity cost per caretaker in the facility was US$21, while that in the community was US$5.8.^11^ A Bangladesh study reiterates that opportunity costs of time and transportation costs in community management are lower than that of facility management.^12^ The CMAM in terms of DALY saved is the fifth most cost-effective intervention (after treatment of malaria, zinc therapy, maternal and neonatal care at home, and micronutrient supplementation) among all the Reproductive, Maternal, Newborn and Child Health (RMNCH interventions identified by Black *et al.*^17^ The same study suggests that interventions costing less than per capita GNI per DALY averted can be termed ‘very cost-effective,’ and those costing less than three times per capita GNI can be termed ‘cost-effective.’ Therefore, at an average cost of US$56 (<3500 rupees) estimated for treating SAM in a community-based setting, the government needs to make treatment of SAM at home a priority, along with actions to prevent SAM.

There is no conclusive evidence to prefer one regimen over the other. The costs of treating with RUTF-C and RUTF-L are almost similar at US$0.4 (23 rupees) per child covered or US$53 for RUTF-C and US$54 for RUTF-L (~3300 rupees) per child treated. The number of weeks of treatment is lowest for RUTF-L, so even with a higher cost of regimen per week per child, the costs for RUTF-L are lowest per covered child. However, the lower number of weeks for RUTF-L does not outweigh the higher regimen costs per week for the treated child, and therefore RUTF-L remains marginally higher than RUTF-C per treated child. In the A-HPF group, food was supplied at 1.5 times the assumed requirements as well as fuel. This also led to the highest cost (US$61) of the A-HPF regimen. This household food sharing can be considered as an additional benefit for a distressed family, leading to 15% higher costs per treated child under A-HPF. The administrative cost under the government programme is approximately 20%, the cost of survey and identification is 10%, the cost of peer support is about 5%, and the cost of treatment varies from 60% to 65% for the three regimens.

Under research activity, the cost per covered child was estimated at US$1.6, and the cost per treated child per episode of treatment was US$227 for RUTF-C, US$229 for RUTF-L and US$238 for A-HPF. Out of these approximately 26%–27% were spent for identification and enrolment, approximately 30%–34% each for treatment and administration, and 9%–10% for peer support.

The total cost for all the 371 treated children under the research programme was estimated at approximately US$85 876 (5324 000 rupees) and almost a quarter at US$20 892 (1295 000 rupees) under the government programme. The administrative costs were approximately 30% in research and around 20% under the government programme.

### Strengths and weaknesses of the study

We did not conduct a full economic analysis or cost-effectiveness analysis because the purpose of the costing analysis was to inform the government about the cost of domiciliary treatment of SAM with the aim of scaling it up. However, we have taken the best possible robust assumptions to derive at the research and government expenses. The strength of the study is in its randomised controlled design, the rigour of implementation of the study, training and standardisation of the staff collecting the costing data, and maintaining specific minute details while tracking costs. The human resource costs have been derived using a time motion type of study, which is more difficult to estimate in a government setting, where the providers are engaged in more than one activity. Further, the costs derived take into account all programmatic and administrative costs, which are more difficult to allocate in the government setting. The limitation of the analysis lies in the extrapolation of research costs to government costs. Ideally, implementing in a government setting would provide a more precise cost estimate.

Second, based on discussions and observations from field visits in Udaipur, Rajasthan and Vellore, Tamil Nadu, we found the costs of interventions identified above varied according to geographical, cultural and programme setting. The differences that would affect the costs were primarily observed in terms of human resource cost and administrative costs to run the programme.

In Udaipur, because of its rural, hilly and widespread (houses away from access roads) terrain, higher transport costs were incurred in actively finding the children with SAM, transporting supplies, and bringing the children to the clinic, further follow-up and referrals. Four-wheel vehicles had to be used to carry the supplies and equipment for weekly follow-up, and long waiting time or several visits had to be made due to unavailability of parents during a visit (as they would be working on fields). Even though salaries are lower in Udaipur, longer time taken by human resources led to higher human resource cost. Larger number of monitoring visits had to be made and ALWs were incentivised based on recoveries. Further the supplies such as ultra-high temperature pasteurised milk had to be arranged from the cities due to unavailability in villages for caste reasons. Connectivity problems led to use of paper forms instead of tablets for recording in Delhi. More intake of contaminated water with RUTF-L and RUTF-C led to diarrhoea in the first fortnight. Families did not tell the staff visiting about diarrhoea to avoid going to clinic. Doctors did not do telephone consultation. Home management was low and referrals were very high. Families were brought to clinics in groups because of large distances. Once brought to clinics, children had to be treated for other morbidities also for ethical reasons, which led to higher costs. Many cases required both home management and hospital treatment. Keeping feeding assistants was a problem due to social reasons.

In Vellore, a semiurban area, the costs seem to be lower. Due to lower SAM prevalence, 55 000 babies were screened to find 282 children with SAM, of whom 252 were enrolled. The lower costs were due to lower salaries in Vellore and lower time taken for different activities. Most deliveries were institutional deliveries and people maintained good record of immunisation and age cards. The distance travelled was from 0.5 km to 35 km, much shorter than in Udaipur. Activities were streamlined and also a nurse was used for several activities, instead of a physician. The costs of RUTF-L preparation and medicines used also seemed to be lower. Medicine procurement was done from internal pharmacy, and mostly generic drugs were dispensed by the pharmacist from the central medical store. The total expenditure on medicine was US$2453 (152 061 rupees) in Vellore compared with US$4285 (265 699 rupees) in Delhi over the same period of 25 months. The costs of RUTF-L worked out lower at approximately US$0.8 (50 rupees) per child per week as compared with US$1.4 (84 rupees) estimated in Delhi for research activity. Home visits and phone consultation were not done and families visited the clinics themselves. Vellore seemed more similar to the government setting, and therefore the costs derived for the government setting in Delhi are likely to be the costs for running the programme efficiently.

### Implications

Our study provides evidence that home-based management of SAM with an RUTF-L is feasible, acceptable, affordable and efficacious. The average costs per treated child in the government setting were estimated at US$56 (~3500 rupees), which can be considered very cost-effective in terms of the DALY saved and GNI per capita of the country. The treatment of SAM at home needs serious attention and integration into the existing health system, along with actions to prevent SAM.

## References

[1] Collins S, Dent N, Binns P, et al Management of severe acute malnutrition in children. Lancet 2006;368:1992–2000. 10.1016/S0140-6736(06)69443-9 17141707

[2] WHO. Management of Severe Malnutrition: A manual for physicians and other senior health workers. Geneva: WHO, 1990.

[3] World Health Organization. Community- Based Management of Severe Acute Malnutrition: A Joint Statement by the World Health Organization, the World Food Programme, the United Nations System Standing Committee on Nutrition and the United Nations Children’s Fund. Geneva: World Health Organization, 2007.

[4] Kapil U Ready to Use Therapeutic Food(RUTF) in the management of severe acute malnutrition in India. Indian Pediatr 2009;46:381–2. 19478351

[5] Ashworth A Efficacy and effectiveness of community-based treatment of severe malnutrition. Food Nutr Bull 2006;27:S24–S48. 10.1177/15648265060273S303 17076212

[6] Bhandari N, Mohan SB, Bose A, et al Efficacy of three feeding regimens for home-based management of children with uncomplicated severe acute malnutrition: a randomised trial in India. BMJ Glob Health 2016;1:e000144 10.1136/bmjgh-2016-000144 PMC5321385 28588982

[7] Gupta P, Shah D, Sachdev HP, et al National workshop on "Development of guidelines for effective home based care and treatment of children suffering from severe acute malnutrition". Indian Pediatr 2006;43:131–9. 16528109

[8] Golden M Questions from the field. Field Exchange, Emergency Nutrition Network 2007;31:17–20.

[9] Prasad V Should India use commercially produced *ready to use th*erapeutic foods (RUTF) for severe acute malnutrition (SAM). Social Medicine 2009;4:52–5.

[10] Sachdev HP, Kapil U, Vir S Consensus Statement National Consensus Workshop on Management of SAM Children through Medical Nutrition Therapy. Indian Pediatr 2010;47:661–5. 10.1007/s13312-010-0097-z 20972283

[11] Tekeste A, Wondafrash M, Azene G, et al Cost effectiveness of community-based and in-patient therapeutic feeding programs to treat severe acute malnutrition in Ethiopia. Cost Eff Resour Alloc 2012;10:4 10.1186/1478-7547-10-4 22429892 PMC3323427

[12] Puett C, Sadler K, Alderman H, et al Cost-effectiveness of the community-based management of severe acute malnutrition by community health workers in southern Bangladesh. Health Policy Plan 2013;28:386–99. 10.1093/heapol/czs070 22879522

[13] Guidelines for ASHA and Mahila Arogya Samiti in the Urban Context. New Delhi: Ministry of Health and Family Welfare, Government of India, 2014.

[14] Bachmann MO Cost-effectiveness of community-based treatment of severe acute malnutrition in children. Expert Rev Pharmacoecon Outcomes Res 2010;10:605–12. 10.1586/erp.10.54 20950075

[15] Wilford R, Golden K, Walker DG Cost-effectiveness of community-based management of acute malnutrition in Malawi. Health Policy Plan 2012;27:127–37. 10.1093/heapol/czr017 21378101

[16] Bachmann MO. 2009. Cost effectiveness of community-based therapeutic care for children with severe acute malnutrition in Zambia: decision tree model. BioMed Central; Cost Effectiveness and Resource Allocation 2009,7: 2. 2009 http://www.resource-allocation.com/content/7/1/2. 10.1186/1478-7547-7-2 PMC2630929 19146668

[17] Black RE, Laxminarayan R, Temmerman M, et al Reproductive, Maternal, Newborn, and Child Health. Disease Control Priorities, third edition, volume 2. Washington, DC: World Bank, 2016. 27227205

